# Thermo-sensitive micelles extend therapeutic potential for febrile seizures

**DOI:** 10.1038/s41392-021-00638-9

**Published:** 2021-08-13

**Authors:** Di Wu, Yangshun Tang, Weishuo Li, Yi You, Jiaying Shi, Cenglin Xu, Yongzhong Du, Zhong Chen, Yi Wang

**Affiliations:** 1 Key Laboratory of Neuropharmacology and Translational Medicine of Zhejiang Province, School of Pharmaceutical Sciences, Zhejiang Chinese Medical University, Hangzhou, China; 2 Institute of Pharmacology and Toxicology, College of Pharmaceutical Sciences, Zhejiang University, Hangzhou, China; 3 Department of Pharmacy, College of Pharmaceutical Sciences, Zhejiang University, Hangzhou, China

**Keywords:** Diseases of the nervous system, Drug delivery

**Dear Editor,**

Febrile seizures (FS) are common convulsive disorder induced by fever, affecting up to 5% of children under the age of 5 years. Although FS are characterized by their benign prognosis, children with complex FS, in the condition with recurrent or prolonged seizures, are at high risks of temporal lobe epilepsy in later life.^1^ Currently, there is no appropriate pharmacotherapeutic option to control FS, and later epileptogenesis in the perspective of both therapeutic efficacy and safety. Therefore, it is significant to understand the mechanism of FS and further to identify potential drug targets for FS treatment. Neuroinflammatory signaling, especially the IL-1β–IL-1R1 pathway, is closely involved in FS and epilepsy.^2^ However, small-molecule inhibitor of IL-1R1 is not available at present. Using pharmacological and genetic intervention, we recently demonstrated that cleaved caspase-1, an IL-1β-converting enzyme, mediates FS generation. After structural virtual screening against the active site of caspase-1, we achieved a novel brain-penetrable small-molecule caspase-1 inhibitor CZL80 “3-(3-(thiophene-2-carboxamido)benzamido)benzoic acid”. The CZL80 could markedly relieve FS generation and later enhance epileptogenic susceptibility with high efficacy.^3^ However, the elimination half-life of the CZL80 is relatively short, which would limit its therapeutic window. Stimuli-responsive “smart” drug delivery carriers, in response to pathological characteristics of disease, have shown great advantages towards biological and biomedical regulation. For example, our previous work demonstrated that electro-responsive hydrogel nanoparticles are able to transport antiepileptic drugs into the brain and release them under electroencephalograph epileptiform abnormalities, which may improve the therapeutic index of existing antiepileptic drugs in clinical use.^4^ As FS is characterized by unpredictable convulsive seizures associated with hyperthermia, we wonder whether hyperthermia could serve as a trigger for drug release in the brain in order to suppress FS in a timely manner. Here, we report a thermo-responsive strategy for efficient FS therapy by loading small-molecule caspase-1 inhibitor CZL80 inside the thermo-sensitive micelles, allowing for drug release upon local heat stimulus and thus extending the therapeutic window for FS (Fig. 1a).

**Fig. 1:**
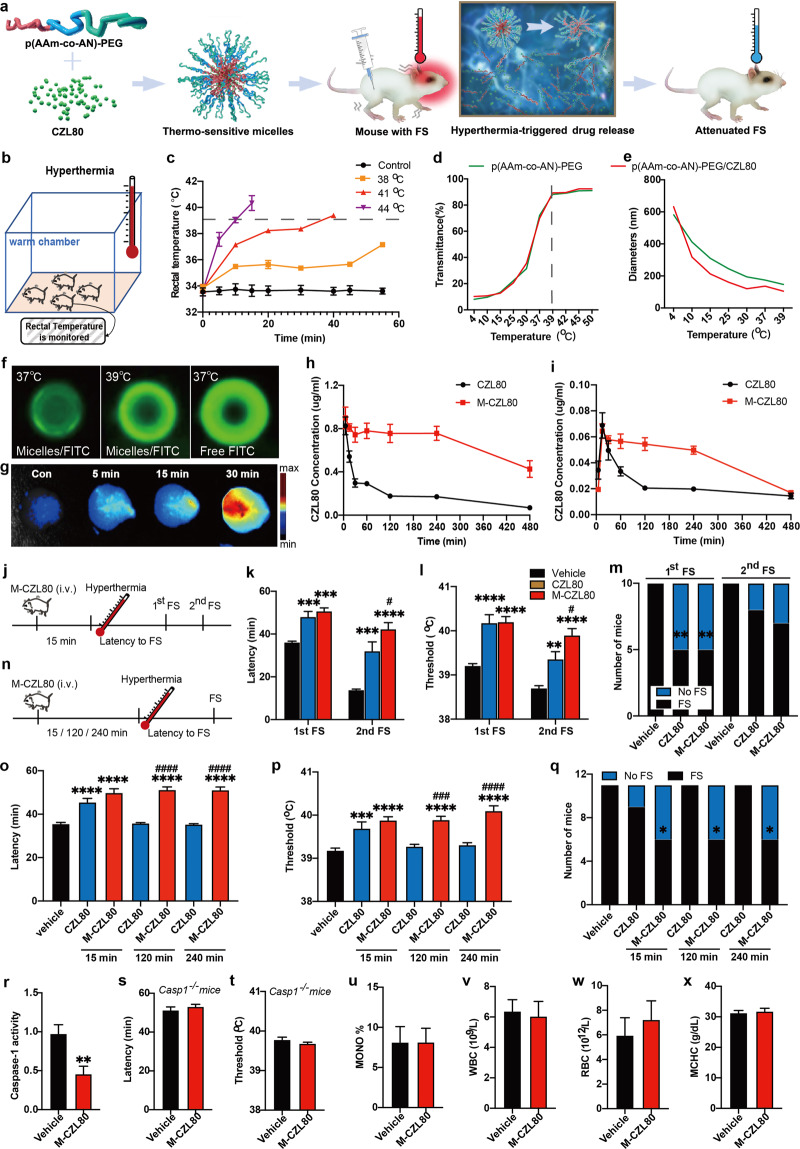
Thermo-sensitive CZL80-loaded micelles extend therapeutic potential in FS model. **a** Experiment scheme of the CZL80-loaded thermo-sensitive micelles (M-CZL80) for the treatment of febrile seizure (FS). **b** Experiment diagram of mouse FS model. P8 mice pups were placed in an incubator chamber with different hyperthermia circumstances (38, 41, or 44 °C) and their rectal temperatures were monitored. **c** Rectal temperatures at different time points for the control and hyperthermia groups. *n* = 10 for each group. **d**, **e** The representative transmittance variations (**d**) and hydrodynamic diameter (**e**) changes of blank micelles (green) and M-CZL80 (red) in response to the temperature shift. **f** Fluorescent images of free FITC and FITC-loaded micelles incubated at 37 or 39 °C for 30 min. **g** Fluorescent images of the brains at different time points after injection (*i.v*.) of micelles. Three individual experiments were replicated. **h**, **i** The concentration of CZL80 in the serum (**h**) and brain (**i**) after the injection (0.75 mg/kg, *i.v*.) of CZL80 and M-CZL80. *n* = 6 for each group. **j** Experiment diagram of M-CZL80 treatment in mouse FS recurrence model. **k**–**m** The latency (**k**), threshold (**l**), and percentage of seizure (**m**) of 1st and 2nd FS after the treatment of vehicle, CZL80 and M-CZL80 (0.75 mg/kg, *i.v*.). *n* = 10 for each group. **k**, **l** One-way ANOVA with Tukey’s post-hoc test; **m** Chi-square test. **P < 0.01, ***P < 0.001, ****P < 0.0001 compared with vehicle group; ^#^*P* < 0.05 compared with CZL80 group. **n** Experiment diagram of M-CZL80 treatment at different time points in mouse FS model. **o**–**q** The latency (**o**), threshold (**p**), and percentage of seizure (**q**) of FS generation after the injection of vehicle, CZL80 (0.75 mg/kg, *i.v*.) and M-CZL80 (0.75 mg/kg, *i.v*.) 15 min, 2 h, and 4 h before the mice were placed in the 41 °C hyperthermia condition. *n* = 11 for each group. **o**, **p** One-way ANOVA with Tukey’s post-hoc test; **q** Chi-square test, *P < 0.05, ***P < 0.001, ****P < 0.0001 compared with vehicle group; ^###^*P* < 0.001, ^####^*P* < 0.0001 compared with CZL80 group. **r** The enzyme activity of caspase-1 in FS mice after the treatment with M-CZL80 (0.75 mg/kg, *i.v*.). *n* = 3 for each group, unpaired *t*-test, **P < 0.01. **s**, **t** The latency (**s**) and threshold (**t**) of FS after the treatment of M-CZL80 (0.75 mg/kg, *i.v*.) in *Casp1*^*−/−*^ mice. *n* = 5 for each group. **u**–**x** The MONO% (**u**), WBC (**v**), RBC (**w**), and MCHC (**x**) were tested after the treatment of M-CZL80 (0.75 mg/kg, *i.v*.). *n* = 5 for each group. Data are presented as means ± s.e.m.

Firstly, the temperature threshold of FS was investigated as a guideline for further thermo-responsive treatment. Mice pups were placed in a hyperthermia chamber at different environmental temperature (38, 41, or 44 °C) and their rectal temperatures were monitored every 5 min, and finally at seizure onset to establish the threshold temperature for FS. (Fig. 1b). We found that FS onset in different hyperthermia conditions occurred when the rectal temperature was above 39 °C (Fig. 1c) and mice would not develop into FS onset with the rectal temperature below 39 °C (Fig. S1), suggesting that 39 °C could be the in vivo seizure-necessity temperature.

Then, the thermo-sensitive micelles of poly(acrylamide co-acrylonitrile)-methoxy polyethylene glycolsuccinimidyl carbonate (p(AAm-co-AN)-PEG) with an upper critical solution temperature (UCST) of 39 °C were synthesized according to our previous study.^5^ The structure of the polymer was confirmed by using ^1^H-NMR (Fig. S2) and FTIR spectroscopy (Fig. S3). When the temperature was lower than 37 °C, the p(AAm-co-AN)-PEG copolymer could self-assemble into defined micelles with a hydrophobic core for cargo loading. Transmission electron microscopy results demonstrated the uniform distribution of the self-assembled micelles (Fig. S4). In contrast, the p(AAm-co-AN)-PEG micelles would disassemble when the temperature reached 39 °C, resulting in a burst drug release. It was indicated that transmittance of the micelle solution increased with the temperature elevation and plateaued when reaching 39 °C (Fig. 1d). These transmittance profiles demonstrated the as-synthesized micelles exhibited a UCST of 39 °C. Size study also showed that hydrodynamic diameter of the micelles decreased when the temperature increased from 4 to 39 °C (Fig. 1e), further proving the micelle had a UCST of 39 °C.

Next, fluorescein isothiocyanate (FITC) was chosen as a model drug to study the thermo-responsive drug release behavior in vitro. The FITC-loaded micelles at 39 °C showed much stronger than that of FITC-loaded micelles at 37 °C (Fig. 1f) due to the aggregation-caused quenching of FITC molecules, while FITC solution featured no difference either after incubation at 37 or 39 °C (Fig. S5). Such fluorescence profiles proved typical thermo-triggered release characteristics of the micelles. Furthermore, indocyanine green (ICG), a near-infrared fluorescent dye was chosen as a model drug to study the drug distribution in the brain in vivo. The micelles quickly accumulated in the brain indicated by the increased fluorescent intensity as early as 5 min (Fig. 1g). This can be due to the incomplete development of blood-brain barrier in mice pups at postnatal day 8. We confirmed this hypothesis by showing the evidence that ICG alone can quickly accumulated in the neonatal brain (Fig. S6). This clearly suggested that the p(AAm-co-AN)-PEG micelles are able to penetrate the blood-brain barrier in neonatal brain, paving the way for brain drug delivery in FS model.

To verify the concept of thermo-responsive therapy of FS, we encapsulated anti-inflammatory small-molecular caspase-1 inhibitor CZL80 into p(AAm-co-AN)-PEG micelles. Thanks to PEGylated micelles, the CZL80 molecules were well protected from being metabolized during blood circulation, and the serum concentration of the CZL80 held a constant concentration for up to 4 h (Fig. 1h). Higher concentration of CZL80 was also found in the brain of micelles groups, lasting for at least 4 h, compared with CZL80 control group (Fig. 1i).

Further, we evaluated the anticonvulsive efficacy of CZL80-loaded micelles in experimental FS model. When injected prior to hyperthermia exposure, the CZL80-loaded micelles prolonged the latency and increased the threshold to the first FS. It also reduced the incidence of first FS to 50% (Fig. 1j–m). Importantly, CZL80-loaded micelles also showed anticonvulsive effects on FS recurrence, which is often resistant to current anti-epileptic drug diazepam. During FS recurrence, it obviously prolonged the latency and increased the threshold to the second FS (better than CZL80 alone), and lowed seizure rate of second FS to 75% (25% showed no second seizure) (Fig. 1k–m). We further compared anticonvulsive efficacy of CZL80-loaded micelles with that of CZL80 alone. We found that CZL80 alone, 15 min (but not 120 or 240 min) after *i.v.* injection, prolonged the latency and increased the threshold to the FS (Fig. 1n–q). This short-window anticonvulsive efficacy of CZL80 is matched with its short elimination half-life time. While CZL80-loaded micelles, 15, 120, or 240 min after *i.v.* injection, all showed anticonvulsive efficacy (Fig. 1o–q). Together, compared with CZL80 alone, the CZL80-loaded micelles improved the anticonvulsive efficacy of CZL80 by extending the therapeutic time-window from 15 min to 4 h, suggesting CZL80-loaded micelles can confer longer-lasting anticonvulsive effect.

Finally, we confirmed that caspase-1 enzyme activity was reduced after the treatment of CZL80-loaded micelles in FS model (Fig. 1r). Importantly, anticonvulsive efficacy of CZL80-loaded micelles was completely lost in FS model with *Casp1*^*−/−*^ mice (Fig. 1s, t), suggesting that caspase-1 activity is required for the anticonvulsive effect of CZL80-loaded micelles. In addition, CZL80-loaded micelles at 10-fold effective concentration did not affect in vitro cell viability (Fig. S7), biochemistry index in in vivo routine blood test (Fig. 1u–x) and the number of neuron and microglia in the cerebral cortex of mice (Fig. S8), suggesting no acute side effect.

In conclusion, we loaded the anti-inflammatory small-molecular caspase-1 inhibitor CZL80 to thermo-sensitive micelles, which permitted drug release upon hyperthermia stimulus and extended the therapeutic window for FS. Seizures can occur at any point during a febrile illness, making it difficult to manage timely. Precise control of drug release under specific pathological temperature during FS is a strategy that appears to address this problem. Thermo-sensitive micelles will rarely release any of the drugs that they are carrying until the body temperature reaches abnormal 39 °C, which may reduce side effects of long-term medication. The micelles can effectively achieve a longer systematic circulation to extend the therapeutic time-window. Meanwhile, it also showed anticonvulsive effects on FS recurrence, which is often resistant to current anti-epileptic drugs. Thus, for those at high risk of seizure under fever, such as people of a young age, people with a low peak threshold or a short latency to seizure, and/or a family history of FS, the CZL80-loaded micelles might be seriously considered for use as an effective and safe prophylaxis to FS generation. This may change the therapeutic paradigm of drug treatment into a type of on-demand “smart” control for FS in the future.

## Supplementary information

Supplementary_Materials
