# Supplementary material for: Thermo-sensitive micelles extend therapeutic potential for febrile seizures
Source: Signal Transduct Target Ther. 2021 Aug 13;6:296. doi: 10.1038/s41392-021-00638-9 (PMC8361119; doi:10.1038/s41392-021-00638-9)
Supplement: Supplementary file 1 — Supplementary_Materials [file 41392_2021_638_MOESM1_ESM.pdf]

# Supplementary Materials for

## Thermo-sensitive micelles extend therapeutic potential for febrile seizures

Di Wu[1,2,a], Yangshun Tang[1,2,a], Weishuo Li[3,a], Yi You[2], Jiaying Shi[2], Cenglin Xu[1], Yongzhong Du[3], Zhong Chen[1*] and Yi Wang[1,2*]

Correspondence to: Professor Zhong Chen (chenzhong@zju.edu.cn); and Professor Yi Wang (wang-yi@zju.edu.cn).

**This PDF file includes:**

Materials and Methods

Figures. S1 to S8

## Materials and Methods

### Animals

Mice pups were the offspring of time-pregnant C57BL/6J and *Casp1*$^{-/-}$ (Caspase-1 knockout) mice. Mothers were maintained in cages with a 12 h light/dark cycle (lights on from 8:00-20:00) and had access to water and food *ad libitum*. The time of birth of pups was monitored every 12 h, and the day of birth was considered postnatal day 0 (P0). All experiments were carried out between 10:00 and 17:00 in accordance with the ethical guidelines of the Zhejiang University Animal Experimentation Committee and the National Institutes of Health Guide for the Care and Use of Laboratory Animals.

### Preparation and characterization of the p(AAm-co-AN)-PEG micelles

p(AAm-co-AN)-PEG micelles was prepared according to our previous report[1]. The chemical structure of p(AAm-co-AN)-PEG polymer was characterized by $^1$H-NMR (400 M, WNMR-I) and FTIR spectroscopy (Nicolet is 50). Then, 10 mg of p(AAm-co-AN)-PEG was dissolved in 5 mL of phosphate buffer (PBS, pH=2.5) and placed at 80 °C for 1 h. The polymer solution was cooled to 35 °C slowly. Subsequently, CZL80 (2 mg/mL, dissolved in DMSO) was added dropwise into a polymeric micelle solution (CZL80: polymers, 10% w/w). Subsequently, the mixture was dialyzed (MWCO: 3.5 kDa) against phosphate buffer (PBS, pH=3) for three changes at an interval of 0.5 h, after that, the dialysis medium was changed into water for another 24 h dialysis, followed by 30 rounds of probe-type ultrasonic treatment (pulsed every 2 s for a 3 s duration). The solution was centrifuged at 4000 rpm for 10 min to eliminate aggregates of non-encapsulated CZL80. After negative staining with 2% $U_2O_2$ acetate solution, transmission electron microscopy (JEM-1200EX, Japan) was conducted for micelle imaging. To further obtain the indocyanine green (ICG)-loaded micelles, tetrabutylammoniumiodide and ICG were co-dissolved in the DMSO (the molecular ratio of tetrabutylammoniumiodide to ICG was 2:1). Ex vivo fluorescent imaging was performed by using IVIS imaging system (Lumina LT, PerkinElmer). Fluorescein isothiocyanate (FITC)-loaded micelles were also fabricated under identical conditions using an identical protocol. Optical transmittance of the solutions at various temperatures was measured at 617 nm using a UV-Vis spectrophotometer (UV-2401, Shimadzu, Japan). Sample cells were thermo-stated with a temperature controller. The heating rate was 1 °C/min. Hydrodynamic diameter of the micelles was determined by DLS using a particle size analyzer (Nano ZS9, Malvern Instruments Ltd., UK). Each analysis was performed in 3 replicates, with a total of 10 runs per replicate at temperatures ranging from 4 °C to 39 °C. The sample was maintained at each temperature for 10 min.

### Evaluation of the thermo-sensitive drug release behaviour of the micelles

Methods to assess the thermal-sensitive drug release behaviour of micelles has been described previously[1]. Briefly, the upper critical solution temperature (UCST) values of the micelles were determined at the temperature at which optical transmittance became constant. Each analysis was performed with 3 replicates, with a total of 10 analyses per replicate, at temperatures ranging from 4 to 50 °C. The samples were maintained at each temperature for 10 min. The

temperature at which the diameter became too small or too large to detect was defined as the UCST. For the *in vitro* tests of the characteristic of temperature-triggered release of micelles, 500 μl of FITC-loaded micelles with a FITC concentration at 16 μg/ml and 500 μl of free recording FITC solution were added to wells in agarose gels. The agarose gels could be prepared by cooling down the hot agarose solution (1.8%) in a 60-mm cell culture dish. Before gelation, a centrifugation tube was placed upside down leaving a hole inside the gel. Then, the agarose gels with free FITC solution or FITC-loaded micelles were then placed at either 37 °C or 39 °C for 0.5 h. Subsequently, an *in vivo* imaging system (CRI Inc., USA) was used to image the agarose gels (excitation: 490 nm, emission: 530 nm). Analysis of CZL80-loaded micelles concentrations in serum and brain tissue was performed as our previous study[2].

**MTT assay**

The cytotoxicities of the CZL80-loaded micelles in Hek293 cells were evaluated using an MTT assay. Briefly, Hek293 cells were seeded in 96-well plates at an initial density of 7,000 cells/well. Cells were exposed to growth medium containing concentrations of the CZL80-loaded micelles (1 or10 μM) at 37°C for an additional 24 h. Then, 150 μL MTT solution (0.5 mg/ml) was added and the cells incubated at 37°C for 2 h, and then the media was replaced with 100 μL DMSO to dissolve the MTT formazan crystals. Plates were shaken for 10 min and the absorbance was measured at 570 nm in a microplate reader (BioRad, Model 680, Hercules, CA).

**Thermo-sensitive micelles treatment in experimental FS model**

Experimental FS were induced in P8 mice pups, who were placed in an incubator chamber with different hyperthermia circumstances (38, 41, or 44°C), as our previous studies[3-5]. Rectal temperature was monitored every 5 min, and finally at seizure onset to establish the threshold temperature for FS. Upon the onset of the first seizure behaviour of falling or tonic-clonic seizures of limbs (1$^{st}$ seizure), the latency to FS was recorded. For recurrent FSs, the pups were moved to a cool surface for 2 min once the 1st seizure was evoked, then returned to the chamber to induce the second seizure (2$^{nd}$ FS). To investigate the effect of CZL80-loaded micelles on experimental FS behaviour, solutions of CZL80 or CZL80-loaded micelles (0.75 mg/kg) were intravenously injected (15, 120 or 240 min) prior to placing the animals into the hyperthermia chambers. The incidence, latency and threshold of 1$^{st}$ and 2$^{nd}$ FS were recorded.

**Caspase-1 activity assays**

Tissues of cerebral cortex in the mice brain were lysed, and caspase-1 activity was assessed using a Caspase-1 Colorimetric Assay Kit (Abcam, ab39470) according to the manufacturer's instruction. The caspase-1 activity was determined by comparing the results of the treatment group sample with those of vehicle group.

**Routine Blood Test**

Routine Blood Test (MONO%, WBC, RBC, MCHC) were measured at 24 h after the treatment of CZL80-loaded micelles (7.5 mg/kg, i.v.) and conducted in Laboratory Animal Center of Zhejiang University. Analysis of CZL80 concentrations in serum and in brain tissue was strictly according to our previous study[2].

**Immunohistochemistry**

Twenty-four hour after the treatment of CZL80-loaded micelles (7.5 mg/kg, i.v.)., mice were deeply anesthetized, and they were perfused with saline and then 4% paraformaldehyde. The brains were removed and stored in 4% paraformaldehyde for 24 h and then in 30% sucrose for 2-3 days. Next, coronal sections were cut with 30-μm thickness on a sliding freezing microtome (CM 3050S, Leica). To identify neuron and microglia, we incubated the brain slices firstly in 0.1% Triton X-100 for 15 min to promote perforation and then in 5% donkey serum for 2 h. After the addition of the primary antibodies for neuronal marker NeuN (MABN140, Millipore) and microglia marker Iba-1 (Abcam, ab178846), the slices were incubated with Alexafluor 488 secondary antibodies. Fluorescent images were collected on a fluorescence microscope (BX61, Olympus, Japan).

**Statistics**

Data are presented as the mean ± s.e.m. Number of experimental replicates (n) and appropriate statistical methods using Prism (version 7.0) are indicated in the figure legends. A two-tailed $P$ value $< 0.05$ was considered statistically significant.

**Data availability**

The datasets generated during and/or analysed during the current study are available from the corresponding author upon reasonable request.

**References:**


1. Li, W., et al. Antitumor drug delivery modulated by a polymeric micelle with an upper critical solution temperature. Angew Chem Int Ed Engl 54, 3126-3131 (2015).
2. Tang, Y., et al. Structure-based discovery of CZL80, a caspase-1 inhibitor with therapeutic potential for febrile seizures and later enhanced epileptogenic susceptibility. Br J Pharmacol 177, 3519-3534 (2020).
3. Wu, D., et al. Intergenerational Transmission of Enhanced Seizure Susceptibility after Febrile Seizures. EBioMedicine 17, 206-215 (2017).
4. Feng, B., et al. Transient increase of interleukin-1beta after prolonged febrile seizures promotes adult epileptogenesis through long-lasting upregulating endocannabinoid signaling. Sci Rep 6, 21931 (2016).
5. Chen, B., et al. Blocking GluN2B subunits reverses the enhanced seizure susceptibility after prolonged febrile seizures with a wide therapeutic time-window. Exp Neurol 283, 29-38 (2016).

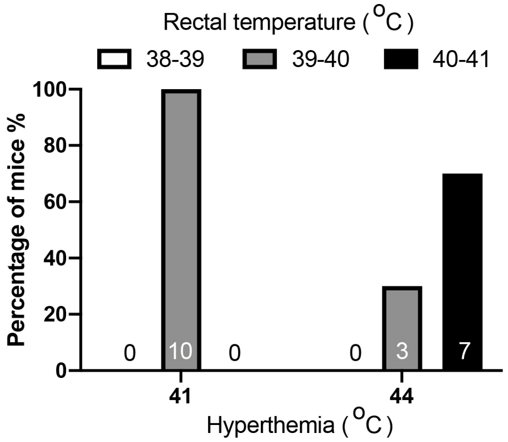


**Fig. S1** Distribution of mice with different rectal temperature of FS onset in 41 ℃ and 44 ℃ hyperthermia conditions.

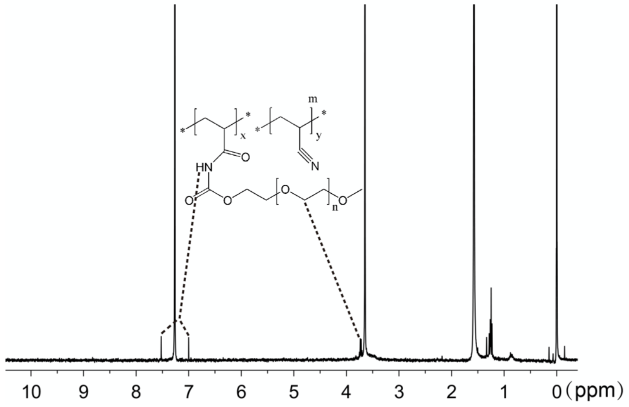


**Fig. S2** The H-NMR spectrum of p(AAm-co-AN)-PEG micelles.

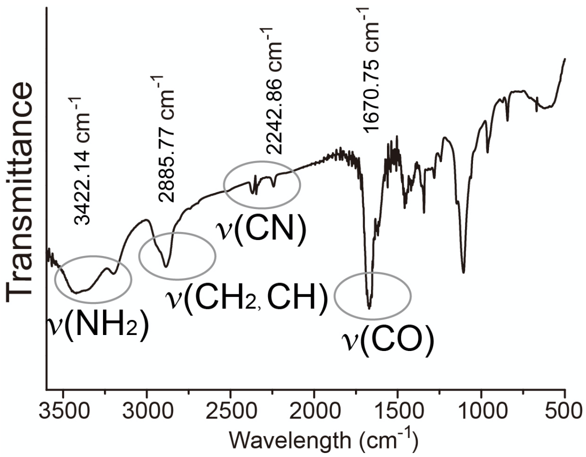


**Fig. S3** The FTIR spectrum of p(AAm-co-AN)-PEG micelles.

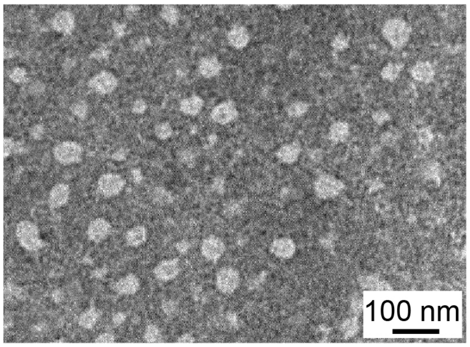


**Fig. S4** The transmission electron microscopy image of p(AAm-co-AN)-PEG micelles.

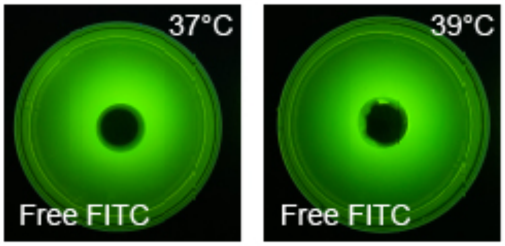


**Fig. S5** Fluorescent images of free FITC in agarose gel incubated at 37°C or 39 °C for 30 min.

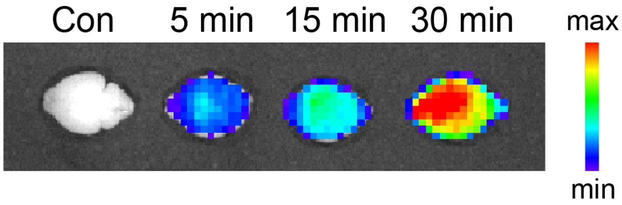


**Fig. S6** Fluorescent image of brains at different time points after the injection (i.v.) of the ICG.

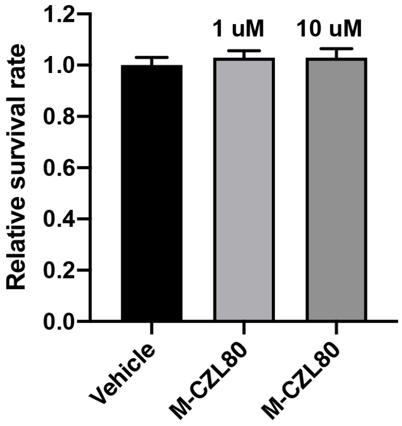


**Fig. S7** Cell viability following incubation of M-CZL80. n=10 for each group. Data are presented as means ± s.e.m..

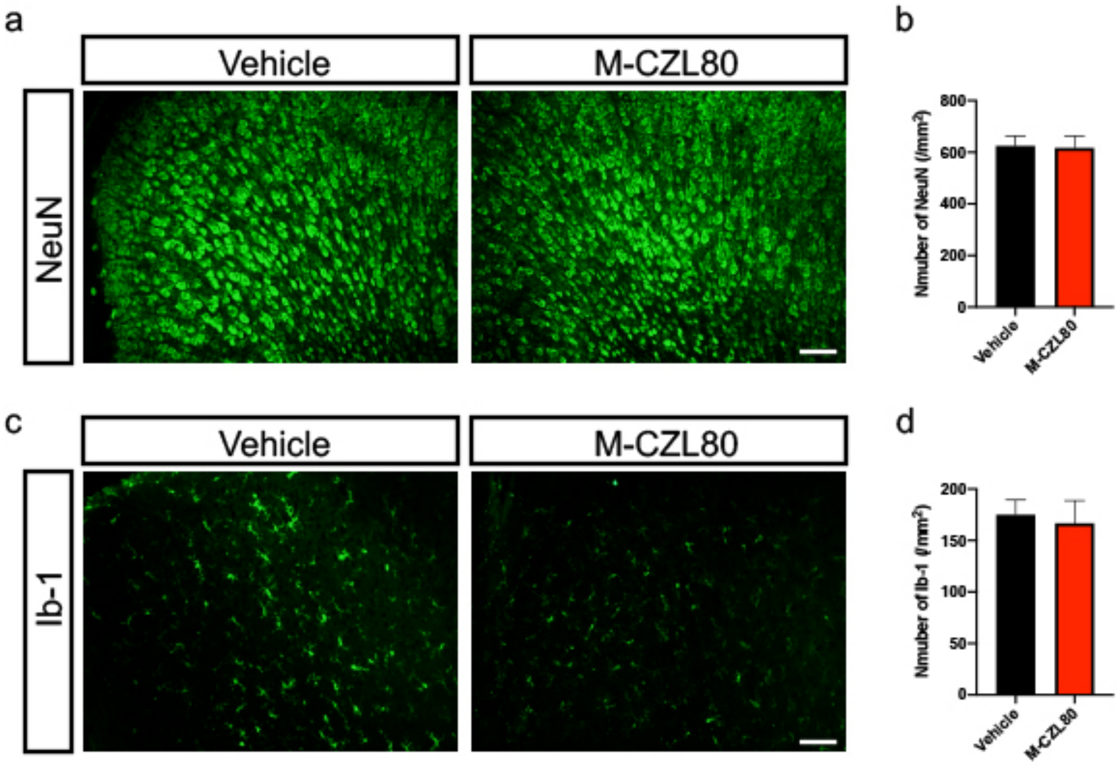


**Fig. S8** Histological analysis of neuron and microglia in M-CZL80-injected mouse brains. (**a, b**) M-CZL80 did not alter the number of neuron (NeuN, a maker for neuron) after the treatment of M-CZL80 (7.5 mg/kg, i.v.). (**c, d**) M-CZL80 did not alter the number of microglia (ib-1, a maker for microglia) after the treatment of M-CZL80 (7.5 mg/kg, i.v.). n=3 for each group. Data are presented as means ± s.e.m..
